# Influence of a Concomitant Medial Meniscus Injury on Knee Joint Function and Osteoarthritis Presence after Anterior Cruciate Ligament Reconstruction

**DOI:** 10.3390/jcm13082433

**Published:** 2024-04-22

**Authors:** Darian Bayerl, Lukas B. Moser, Markus Neubauer, Johannes Neugebauer, Dietmar Dammerer, Markus Winnisch, Rudolf Schabus

**Affiliations:** 1 Department for Orthopedics and Traumatology, Karl Landsteiner University of Health Sciences, 3500 Krems, Austria; 2 Division of Orthopaedics and Traumatology, University Hospital Krems, Mitterweg 10, 3500 Krems, Austria; 3 Medical Practice Sport&Trauma, Pelikangasse 15 A/5, 1090 Vienna, Austria; 4 Center for Regenerative Medicine, Danube University Krems, 3500 Krems, Austria

**Keywords:** anterior cruciate ligament, ACL, ACL rupture, ACL repair, meniscus injury, osteoarthritis, KOOS, IKDC, Lysholm Score, Tegner Activity Score

## Abstract

**(1) Background:** The aim of this study was to investigate how a medial meniscus injury accompanying an anterior cruciate ligament rupture affects the clinical outcome 10 years after ACL reconstruction. **(2) Methods:** A total of 37 patients who received anterior cruciate ligament reconstruction (ACLR) were included in this retrospective study. Two groups were analyzed at a single follow-up of 10 years: (i) “isolated (ACLR)” (*n* = 20) and (ii) “ACLR with medial meniscal injury” (*n* = 17). The following clinical scores were recorded: International Knee Documentation Committee (IKDC), the Knee Injury and Osteoarthritis Outcome Score (KOOS), Lysholm Score and Tegner Activity Score. To determine the degree of osteoarthritis the Kellgren–Lawrence score was used. **(3) Results:** The “isolated ACLR” study group scored significantly higher (*p* < 0.05) on the IKDC subjective questionnaire (mean: 88.4) than the “ACLR with medial meniscus injury” group (mean: 81). The KOOS category “activities of daily living” showed significantly better results in the isolated ACLR group (*p* < 0.05). The “ACLR with medial meniscus injury” group had significantly higher degree of osteoarthritis (*p* < 0.05). No significant differences were found in all the other clinical scores. **(4) Conclusions:** The results of this study further indicate that patients with a concomitant medial meniscus injury have slightly more discomfort in everyday life and increased risk of developing osteoarthritis 10 years after surgery.

## 1. Introduction

Every second anterior cruciate ligament injury is accompanied by an meniscal injury [1]. The ruptured ACL leads to instability and a change of loading distribution in the knee joint. This results in increased forces acting on the meniscus and cartilage, which can ultimately lead to its damage and promote the development of osteoarthritis [2]. Biomechanical studies have shown that a ruptured ACL leads to an increase in contact forces during anterior tibial translation by a factor of 4. This leads to a significantly increased ring tension on the medial meniscus, of which the posterior horn is mostly affected. In the event of a cruciate ligament injury, the posterior horn of the medial meniscus takes on a role as a secondary stabilizer to mechanically counteract the anterior translation, which leads to increased stress on the meniscus and promotes the occurrence of a meniscal injury. Gupta et al., reported that in patients with a deficient anterior cruciate ligament, meniscal tears that occupy more than 40% of the width of the medial posterior horn lead to increased instability. According to Arno et al., meniscectomies of more than 46% of the width of the medial posterior horn also lead to increased knee instability [3,4,5]. The clinical impact of concomitant meniscal injury after a short follow-up period (<5 years) is controversial. Some studies suggest that meniscal injury has no effect two years after surgery [6,7,8] whereas other studies show a negative effect on clinical outcome [9]. However, the influence of a concomitant meniscal injury on the onset and progression of osteoarthritis has already been demonstrated [10,11]. Especially, a meniscectomy has a negative effect on the development of osteoarthritis, while meniscus preservation has been proven to be beneficial. A total or partial meniscectomy leads to a reduction in stability and contact area in the knee joint, which consequently increases the point load in the joint. This stress is in turn associated with increased biochemical processes and cartilage deformation, possibly leading to osteoarthritis [12]. Above all, the extent of the meniscectomy is a decisive factor—the more meniscus is damaged and has to be removed, the more severe degenerative joint changes are to be expected [13]. However, the clinical and radiological effects of an ACL reconstruction accompanying meniscal injury (>10 years) have not yet been sufficiently investigated so far. The aim of this study is to investigate the influence of a concomitant medial meniscus injury in ACL reconstruction after a long-term follow-up of 10 years. The hypothesis is that there is no clinical and radiological difference between isolated ACL reconstruction and ACL reconstruction with a meniscal injury after 10 years.

## 2. Materials and Methods

Subsequently, the records of all surgical reports (period 1 January 2008 to 31 December 2009) were searched in the surgery database of the surgery “Sport & Trauma” (Wiener Privatklinik, Pelikangasse 15, 1090 Vienna, Austria)—(surgery database Filemaker 16^®^—Claris International Inc.^®^, Cupertino, CA, USA) for patients who underwent ACL reconstruction. The injuries were verified by MRI. All surgeries were performed arthroscopically by the same experienced surgeon, RS, using a semitendinosus tendon (SST). Meniscectomies were performed using a shaver and punch and meniscus sutures using the all-inside technique. With regard to rehabilitation, a standard procedure was applied. Patients with a medial meniscus injury were treated no differently than patients with an isolated cruciate ligament injury, with the exception of patients with a meniscus suture, who were given 4 weeks of unloading. The surgical reports were then reviewed according to the inclusion and exclusion criteria. The following patients were included in the study (i) written informed consent for follow-up available; (ii) age at the time of injury >18 and <70 years; (iii) division of patients into 2 groups: isolated ACL without concomitant meniscal injury (group 1) and ACL with concomitant meniscal injury (group 2). The following patients were excluded: (i) age at the time of injury <18 and >70 years; (ii) previous surgery on the affected limb; (iii) no complete ACL rupture; (iv) concomitant injury of the knee ligamentous apparatus; (v) pregnancy at the scheduled follow-up.

**Figure 1:**
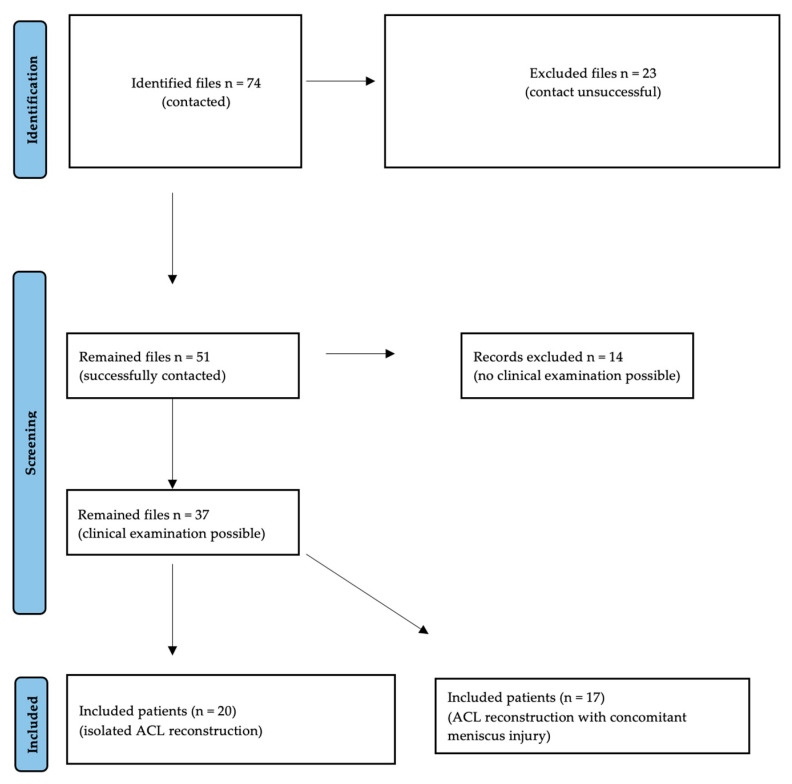
Selection Process of the Study groups.

A total of 74 patients met the inclusion criteria. All patients were contacted and invited for clinical and radiological follow-up examinations. A total of 51 patients were successfully contacted, 37 of whom attended the follow-up examination. A total of 20 patients underwent an isolated ACL reconstruction group (group 1), whereas 17 patients underwent ACL reconstruction with concomitant meniscus injury (group 2) (Figure 1).

**Follow-up examinations**

The following clinical scores were recorded after a follow-up of 10 years after surgery: International Knee Documentation Committee (IKDC), the Knee Injury and Osteoarthritis Outcome Score (KOOS), Lysholm Score, and Tegner Activity.

An objective assessment of the knee joint was carried out using the “IKDC—Knee Examination Form”. The test comprises seven main groups (effusion, passive motion deficit, ligament examination, compartment findings, harvest site pathology, X-ray findings, and functional test), which can be broken down into additional subgroups. Both the subgroups and the main groups are determined based on four grades (A, B, C, D), with grade A corresponding to normal findings and grade D to clearly abnormal findings. The degree of the main group depends on the lowest grade within a group. The final assessment results from the worst grade of the first three main groups [14]. The IKDC form for the subjective assessment of the knee consists of three categories (“symptoms”, “sports activities”, and “function”) with a total of 18 questions. This test can be used to measure subjective improvements or deteriorations in the respective categories in patients with knee injuries. The points achieved are added up at the end and standardized on a scale ranging from 0–100 (percentage of the possible total score). A score of 100 means symptom-free and indicates that there are no restrictions on daily and sporting activities [15]. The KOOS test was first published in 1998 and is used to evaluate the short and long-term consequences of knee injuries and osteoarthritis. It comprises five categories (“symptoms”, “pain”, “activities of daily living”, “sport and recreation function”, and “knee-related quality of life”) with a total of 42 questions. For each question, there are five possible answers corresponding to a score from 0 to 4. The results are added up for each category and finally, with the help of a special formula, are converted to a scale ranging from 0 to 100 (percentage of the possible total score). A value of 0 indicates extreme knee problems, and a value of 100 means that there are no knee problems [15,16].

The Lysholm Score was first published in 1982 by Lysholm and Gillquist and is used to examine the functional impairment of the patient due to subjective clinical instability [17]. After a modification in 1985, it comprises finally, eight questions relating to complaints and abilities in the categories “limp”, “support”, “locking”, “instability”, “pain”, “swelling”, “stair climbing”, and “squatting”. These should be answered by the patient. A maximum of 100 points can be achieved, whereby a score of 100 means that the patient has no symptoms. A score of 95 to 100 is rated as excellent, a score of 84 to 94 as good and a score of 65 to 83 as moderate. If less than 65 points are achieved, this corresponds to a poor result [15,18]. The Tegner Activity Scale was used to support the modified Lysholm Score. It consists of a list of activities ranging from restricted walking and disability to recreational sports to competitive sports at national and international level. The different activity levels are listed on a scale from 0 to 10. The patient selects the level that best reflects their current activity level [15,18]. In addition to the clinical scores, X-rays of the affected knee joint were taken at a nearby X-ray institute (Rosenberg image, anterior–posterior (a.p.), and lateral image (l.) in standing position, patella tangential image). The degree of osteoarthritis was determined according to the Kellgren–Lawrence score with regard to the criteria of osteophyte formation, joint space reduction, sclerosis, and deformation of the joint-forming bone parts [19].

**Statistical analysis**

Demographic data such as age and gender were summarized using descriptive statistics. Chi-square tests were used to compare the scores. The level of significance was defined as *p* < 0.05. Metric variables were tested for normal distribution using the Shapiro–Wilk test and checked for homogeneity of variance using Bartlett’s test. The distributions of metric variables showed a significant deviation from a normal distribution. The variances were homogeneous in each case. These data were therefore presented as medians and quartiles, and the Kruskal–Wallis test (KW test) was applied. The data collected were analyzed using the statistical software R^®^ (version 4.0.0).

## 3. Results

Of the 37 study participants, 16 (43.24%) were male and 21 (56.76%) were female. The study group “isolated ACLR” consisted of 20 participants (30% male, 70% female), and the study group “ACLR with medial meniscus injury” consisted of 17 participants (58.8% male, 41.2% female). The youngest participant was 28 years old, the oldest 70 years old. The lightest and heaviest participants weighed 43 kg and 110 kg. In the “isolated ACLR” group, the average age was 47 ± 9.1 years, and the average weight was 77.2 ± 15.0 kg; in the “ACLR with medial meniscus injury” group, the average ages were 54.82 ± 11.8 years and 81.2 ± 16.9 kg.

The average age on the day of examination was 48.8 ± 11.8 years for the entire collective. On average follow-up was carried out 3978 days (130 months) ± 211 days (about 7 months) after surgery. In 15 of 17 cases (88.2%) the meniscus had been treated by partial meniscectomy, in two cases (11.7%) a meniscal suture had been performed as treatment.

**IKDC**

No significant difference was found in the objective IKDC score between the two study groups. The distribution of the degrees of completion can be seen in Table 1.

No patient complained of irritation/numbness at the donor site of the autologous transplant. No patient had a passive movement deficit. The distributions of the individual categories can be seen in Table 2.

**Figure 2:**
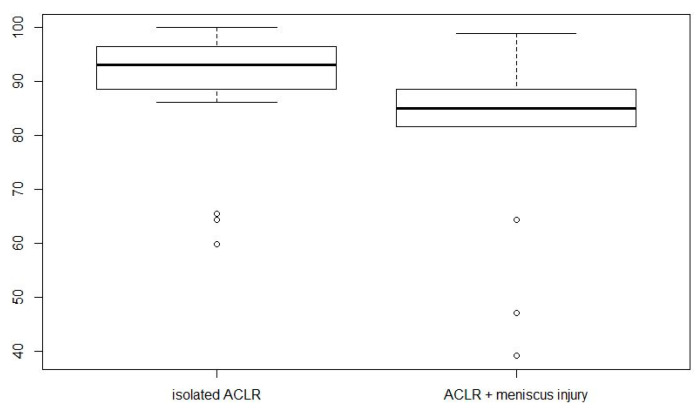
Results of subjective IKDC questionnaire.

There was a significant difference (*p*-value: 0.02) in the subjective IKDC score between the isolated ACLR group (88.4 ± 13.0) and the “ACLR with medial meniscus injury” (81 ± 16.1) (Figure 2).

**KOOS**

Patients with isolated ACL reconstruction scored significantly (*p*-value: 0.045) higher in the “activities of daily living” category than the comparison group (Table 3). All other categories (“symptoms”, “pain”, “sports and recreation function”, and “knee-related quality of life”) showed no significant difference between the study groups.

**Tegner Activity Scale**

All patients had a Tegner Activity Scale > 2 (Table 4). There was no significant difference between the two study groups. More than half of both study groups reported an activity level of ≥ level 5 (65% of isolated ACL reconstruction and 59% of ACL reconstruction with medial meniscus injury). This is at least equivalent to performing vigorous physical work, cycling, cross-country skiing competitively, and jogging on uneven surfaces > 2/week.

**Lysholm Score**

**Figure 3:**
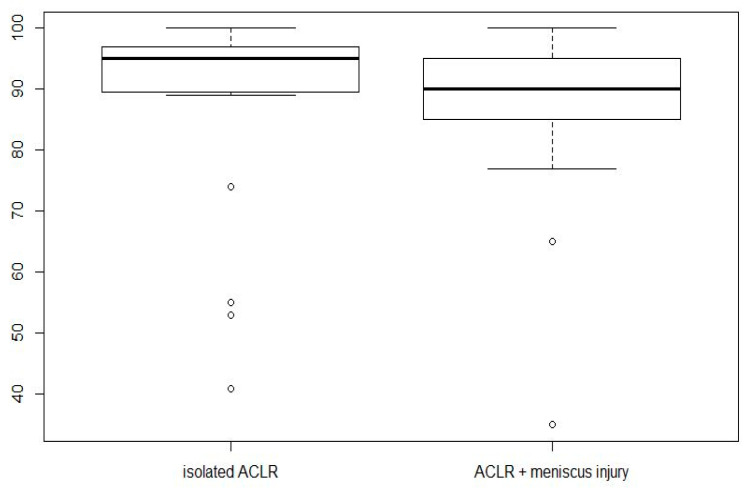
No significant difference between the two groups concerning the Lysholm score.

The Lysholm score did not differ significantly between the two groups (Figure 3). The mean value was 87.3 ± 17.4 in the “isolated ACLR” group and 86.7 ± 16 in the “ACLR with medial meniscus injury” group.

**Kellgren–Lawrence score**

The group with concomitant medial meniscus injury showed significantly higher KL scores than the comparison group (Table 5).

## 4. Discussion

The most important findings of the present study are the following:

Firstly, ACL reconstruction showed satisfying clinical outcome in both groups after a long-term follow-up 10 years. Objective assessment of the knee joint using IKDC showed no significant difference with good results for both groups. 85% of the “isolated ACL reconstruction” group had grade A or B which corresponds to a normal or nearly normal joint. In group with medial meniscus injury even 94% had grade A or B. Shelbourne and Gray had slightly lower values for the group with a meniscal injury in their follow-up examination 5–15 years postoperatively: 87% of patients with a healthy meniscus had grade A or B, and only 63% with partial or total medial meniscectomy had grade A or B [13]. In the present study, the median of all KOOS categories (except the “knee-related quality of life” category in the “isolated ACLR” group) was at least 90 in both study groups. This means that more than half of all subjects had few to no knee problems. In the Lysholm score, the mean value in the “isolated ACL reconstruction” group was 87.3 and in the “ACL reconstruction with medial meniscus injury” group it was 86.7. In the Lysholm score, this number of points means excellent knee function. In the Tegner Activity Scale, no patient had an activity level <3, which means that recreational sports activities such as swimming, hiking, or walking are possible. More than half of both study groups (65% and 59%, respectively) even had an activity level of ≥5, which corresponds to activities such as skiing, ski touring, cycling, gymnastics, gymnastics, and jogging.

Secondly, patients with ACL reconstruction with concomitant meniscal injury performed significantly worse in the subjective IKDC (88.4 vs. 81) and in the KOOS category “activities of daily living” than patients with intact menisci. These results are partly supported by the literature. A registry study from Sweden with more than 10,000 patients included has shown that after a follow-up of 2 years an ACL reconstruction accompanied by meniscectomy was associated with a worse outcome than an isolated ACL reconstruction. In the comparison group of concomitant meniscal refixation, an outcome comparable to isolated ACL reconstruction was found [20]. Wu et al., came to a similar conclusion in a longer follow-up after 10 years but with a significantly smaller patient population (63 patients). Patients with a meniscectomy at the time of ACL reconstruction had significantly more subjective complaints than patients whose menisci were repaired. The authors concluded that the meniscus should always be preserved if possible [21]. Another large registry study from Sweden (>1000 patients) showed a worsening of the clinical outcome in the group with concomitant meniscal injuries after 5 to 10 years in the KOOS categories “pain”, “symptoms”, “sports and recreation function”, and “knee-related quality of life”. No deterioration was observed in patients with isolated anterior cruciate ligament ruptures [9]. Other studies, however, showed no influence of concomitant meniscal injury on individual clinical scores. Røtterud et al., did not find an influence of concomitant meniscal injury on the KOOS in a Norwegian registry study with 8476 included patients after a follow-up of 2 years [6]. Spindler et al. also found no influence of an accompanying meniscus injury on the KOOS, the Lysholm score, or the subjective results of the IKDC questionnaire after 5 years of follow-up. Likewisem Kowalchuk et al., found that meniscal injury during ACL reconstruction was not a predictor of poorer outcomes on the IKDC subjective questionnaire. This is in contrast to the present study where significant differences in the IKDC questionnaire were found [7,8].

Thirdly, the study showed that a medial meniscus injury accompanying an ACL injury has a negative effect on the presence of osteoarthritis. These results are consistent with the available literature. Porat et al. examined 219 male soccer players 14 years after ACL reconstruction and were able to show that a concomitant meniscus injury leads to increased development of osteoarthritis. 59% of patients with ACL rupture and concomitant meniscal injury showed a KL score ≥ 2, whereas only 31% of participants with isolated anterior cruciate ligament injury were assessed with a grade of ≥2 [11]. Similarly, in the present study 71% of the group “ACL with medial meniscus injury” had a KL-Score ≥grade 2, and only 25% of the group “isolated ACL” radiologically ≥2. Lohmander et al., examined 103 female soccer players 12 years after ACL injury and showed that patients with additional meniscus surgery had a higher prevalence of osteoarthritis (69% vs. 39%) [10]. However, no distinction was made according to the Kellgren–Lawrence score. The decisive factor for the occurrence of post-traumatic osteoarthritis after cruciate ligament reconstruction is whether the meniscal injury was treated by meniscectomy or whether an attempt was made to preserve the meniscus using meniscal refixation. Nakata et al., examined 61 athletically active patients 10 years after ACL reconstruction and were able to show that 13/15 patients (87%) with additional meniscectomy showed degenerative joint changes. In the comparison group (patients without meniscus injury or meniscus refixation), only 12/46 patients (16%) were found to have osteoarthritis [22]. Wu et al., showed similar results in their study. 10 years after surgery, all 9 patients who had undergone an total meniscectomy at the time of cruciate ligament reconstruction showed radiographic evidence of osteoarthritis. In contrast, 23 out of 25 (92%) patients who had intact menisci showed no degenerative changes [21]. Aglietti et al., also saw more degenerative joint changes in the study group with partial meniscectomies than in the group with meniscus refixations [23]. Shelbourne and Gray also showed the negative influence of meniscectomies on the development of osteoarthritis. On average 8 years after the operation, the different study groups were assessed radiologically using IKDC. The participants whose menisci were both intact performed best. Here, 97% received group grade A or B and 3% received group grade C or D. In the group with lateral meniscectomy, 9% received group grade C or D and in the group with medial meniscectomy, 23% received group grade C or D. The group in which both a medial and a lateral meniscectomy had been performed fared the worst. Here, 25% were radiologically graded C or D [13]. In the present study, the knee joint was also assessed radiologically using IKDC. Group grade A or B was given to 85% in the “isolated ACLR” group and 71% in the “ACLR with medial meniscus injury” group. The remaining 15% and 29% were graded group C. In the event of a cruciate ligament injury, there is an increased release of local inflammatory mediators, so-called cytokines (TNF-alpha, interleukin-1β, and interleukin-6). These inflammatory mediators can have a negative effect on the integrity of the cartilage, and a post-traumatic inhibition of chondrogenesis is being discussed. Interestingly, the subsequent cruciate ligament reconstruction also leads to a release of these cytokines into the knee joint [2]. Especially in the presence of cartilage damage, the concentration of chondrodestructive cytokines is further increased depending on the extent of the defect: the larger the defect, the higher the cytokine concentration [24]. Ichiba et al., were able to show the effects of cartilage damage for ACL reconstruction on osteoarthritis progression in a clinical study involving 46 patients. Patients with cartilage damage had a significant increase in KL score compared to the group without cartilage damage [25].

The present study has some limitations. The retrospective study design relied on existing data, which can be subject to biases and confounding variables. The patient population was small with 37 patients, which may have led to the fact that no statistically significant difference could be shown in some subjective scores. All objective examinations are examiner-dependent and were performed by one examiner. The assessment of the KL score is subject to high intra-rater and inter-rater variability. Studies have shown that an AI-based assessment provides more reliable results [26]. Furthermore, the type and location of the meniscal injury was not documented during surgery and there was no intraoperative assessment of pre-existing osteoarthritis. In the group with meniscal injuries, two patients underwent meniscal suturing. Even if the proportion of the total group is low (12%), this could have influenced the results. With regard to osteoarthritis, it is also a limitation that no X-rays were taken preoperatively to determine the KL score. This would have made it possible to assess the progression of osteoarthritis, which is not possible with postoperative determination alone.

## 5. Conclusions

Patients with isolated ACLR and patients with ACLR plus concomitant medial meniscus injury have a satisfactory clinical outcome 10 years postoperatively. The results of this study indicate that patients with a concomitant medial meniscus injury have slightly more discomfort in everyday life and increased risk of developing of osteoarthritis 10 years after surgery.

## Figures and Tables

**Table 1:** IKDC final assessment—absolute and relative frequencies.

Degree	Isolated ACL Reconstruction	ACL Reconstruction + Meniscus Injury	*p*-Value
A	8 (40%)	8 (47%)	0.92
B	9 (45%)	8 (47%)	1
C	3 (15%)	1 (6%)	0.44
D	0	0	-

**Table 2:** IKDC group grades—absolute and relative frequencies.

	Isolated ACL Reconstruction				ALC Reconstructtion + Meniscus Injury			
Group Grade	A	B	C	D	A	B	C	D
Effusion	18 (90%)	2 (10%)	0	0	15 (88%)	2 (12%)	0	0
Passive motion deficit	20 (100%)	0	0	0	17 (100%)	0	0	0
Ligament examination	9 (45%)	8 (40%)	3 (15%)	0	9 (53%)	7 (41%)	1 (6%)	0
Compartment findings	6 (30%)	13 (65%)	1 (5%)	0	6 (35%)	9 (53%)	2 (12%)	0
Harvest site pathology	20 (100%)	0	0	0	17 (100%)	0	0	0
X-ray findings	3 (15%)	14 (70%)	3 (15%)	0	2 (12%)	10 (59%)	5 (29%)	0
Functional test	18 (90%)	2 (10%)	0	0	14 (82%)	2 (12%)	0	1 (6%)

**Table 3:** This table shows the results of the KOOS questionnaire for both groups.

Dimension	Isolated ACLR		ACLR + Meniscus Injury		*p*-Value
	MW	SD	MW	SD	
Symptoms	91.6	12.4	91.4	11.9	0.706
Pain	93.3	12.0	88.4	16.0	0.144
Activities of daily living	96.9	6.7	92.7	11.2	0.045 *
Sports and recreation function	88.5	20.1	80.9	23.3	0.128
Knee-related quality of life	75.9	25.8	78.7	24.3	0.699

* statistically significant *p* < 0.05.

**Table 4:** This table shows the absolute and relative frequency distribution of the activity level of both groups (Tegner Acvitity Scale).

Level	Isolated ACL Reconstruction	ACL Reconstruction + Meniscus Injury	*p*-Value
0	0	0	-
1	0	0	-
2	0	0	-
3	1 (5%)	3 (18%)	0.32
4	6 (30%)	4 (24%)	0.73
5	5 (25%)	3 (18%)	0.70
6	2 (10%)	0 (0%)	0.49
7	6 (30%)	6 (35%)	1
8	0	0	-
9	0 (0%)	1 (6%)	0.46
10	0	0	-

**Table 5:** Absolute and relative frequencies of KL scores.

	Isolated ACL Reconstruction	ACL Reconstruction + Meniscus Injury	*p*-Value
Grade 1	15 (75%)	5 (29%)	0.015 *
Grade 2	3 (15%)	10 (59%)	0.015 *
Grade 3	2 (10%)	2 (12%)	1
Grade 4	0	0	-

* statistically significant *p* < 0.05.

## Data Availability

Data available on request due to restrictions, e.g., privacy or ethical. The data presented in this study are available on request from the corresponding author.
